# Improved NMDA Receptor Activation by the Secreted Amyloid-Protein Precursor-α in Healthy Aging: A Role for D-Serine?

**DOI:** 10.3390/ijms232415542

**Published:** 2022-12-08

**Authors:** Jean-Marie Billard, Thomas Freret

**Affiliations:** UNICAEN, INSERM, COMETE, CYCERON, CHU Caen, Normandie Université, 14000 Caen, France

**Keywords:** amyloid protein precursor, hippocampus, electrophysiology, glutamate, synaptic plasticity, long-term potentiation, serine racemase

## Abstract

Impaired activation of the N-methyl-D-aspartate subtype of glutamate receptors (NMDAR) by D-serine is linked to cognitive aging. Whether this deregulation may be used to initiate pharmacological strategies has yet to be considered. To this end, we performed electrophysiological extracellular recordings at CA3/CA1 synapses in hippocampal slices from young and aged mice. We show that 0.1 nM of the soluble N-terminal recombinant fragment of the secreted amyloid-protein precursor-α (sAPPα) added in the bath significantly increased NMDAR activation in aged but not adult mice without impacting basal synaptic transmission. In addition, sAPPα rescued the age-related deficit of theta-burst-induced long-term potentiation. Significant NMDAR improvement occurred in adult mice when sAPPα was raised to 1 nM, and this effect was drastically reduced in transgenic mice deprived of D-serine through genetic deletion of the synthesizing enzyme serine racemase. Altogether, these results emphasize the interest to consider sAPPα treatment targeting D-serine-dependent NMDAR deregulation to alleviate cognitive aging.

## 1. Introduction

Among the wide range of neurotransmitter receptors expressed in the nervous system, the N-methyl-D-aspartate glutamate receptors (NMDARs) certainly hold a prominent and original position in view of the numerous functions they regulate and the mechanisms governing their activation [1]. Notably, NMDARs are key to functional synaptic plasticity in neuronal networks such as long-term potentiation (LTP) underlying learning and memory as well as to mechanisms driving neurotoxicity [2,3]. As such, they have been linked to the development of numerous neurological and neurodegenerative diseases and have also been associated with cognitive degradation occurring in healthy aging (reviewed in [4,5,6,7]). In fact, a wealth of preclinical evidence indicates that decreased expression and/or dysfunction of NMDARs underlie memory deficits and long-term potentiation (LTP) impairment that usually take place at an advanced age [6,8,9,10,11,12]. In addition to glutamate, NMDARs require the binding of a coagonist for channel opening [13,14]. Today, preclinical studies indicate that the amino acid D-serine synthesized from L-serine by serine racemase (SR) is the main endogenous NMDAR coagonist in cognitive-related brain structures [15,16,17,18,19]. Decrease in hippocampal D-serine levels inducing NMDAR hypoactivation is now viewed as key mechanism underlying age-related memory impairment and is therefore considered an interesting target to alleviate cognitive aging [20,21,22,23,24,25]. Due to adverse effects [26,27,28,29,30], D-serine chronic treatment is questioned [31,32], and alternative strategies aimed at increasing endogenous levels of the coagonist have thus been initiated such as inhibition of D-amino acid oxidase (DAO), the major catabolic clearance pathway of the amino acid [33,34,35,36,37]. Although a number of DAO inhibitors have been developed, nonconclusive results have yet been reported in preclinical studies in terms of improvement of hippocampal functionality and D-serine availability [38,39,40,41], and most of the compounds have not entered clinical trials. Acting through the facilitation of D-serine production therefore appears as a necessary alternative [20] that implies the characterization of active molecules with minimal adverse effects. Among several putative candidates, the secreted amyloid protein precursor-α (sAPPα) generated from proteolysis of the amyloid protein precursor through the non-amyloidogenic pathway is viewed as being a promising therapeutic molecule [42]. In addition to neurotrophic and neuroprotective properties [43,44,45], sAPPα prevents memory deficits in aged rats [46,47] as well as in different Alzheimer’s mouse models [48,49,50,51,52,53]. However, it remains to be established whether sAPPα also rescues LTP deficit in aged mice, whether NMDARs contribute and what the underlying molecular mechanisms are ([42,47,54,55] but see [56]). Notably, whether D-serine is involved could be asked since prolonged application of sAPPα was found to promote SR expression and D-serine release in hippocampal microglial cell cultures [57].

In the present study, we show at CA3/CA1 synapses of mouse hippocampal slices that bath application of 0.1 nM sAPPα significantly facilitates NMDAR activation in aged but not in adult mice and is thus able to rescue age-associated LTP deficit. In parallel, we report that raising sAPPα concentration to 1 nM also significantly enhances NMDAR activation in adult animals, and we find that this improvement is severely reduced in mice genetically deprived of SR (SR-KO), the D-serine synthesizing enzyme whose function is known to be altered in aging [4,11,23,58]. Taken together, these results suggest that SR could be one privileged target for sAPPα to rescue age-related functional synaptic NMDAR deregulation and confirm the pivotal role of D-serine and its synthesizing enzyme to develop innovative pharmacological strategies aimed at preventing cognitive aging [59].

## 2. Results

### 2.1. Isolated NMDAR Activation

**Figure 1:**
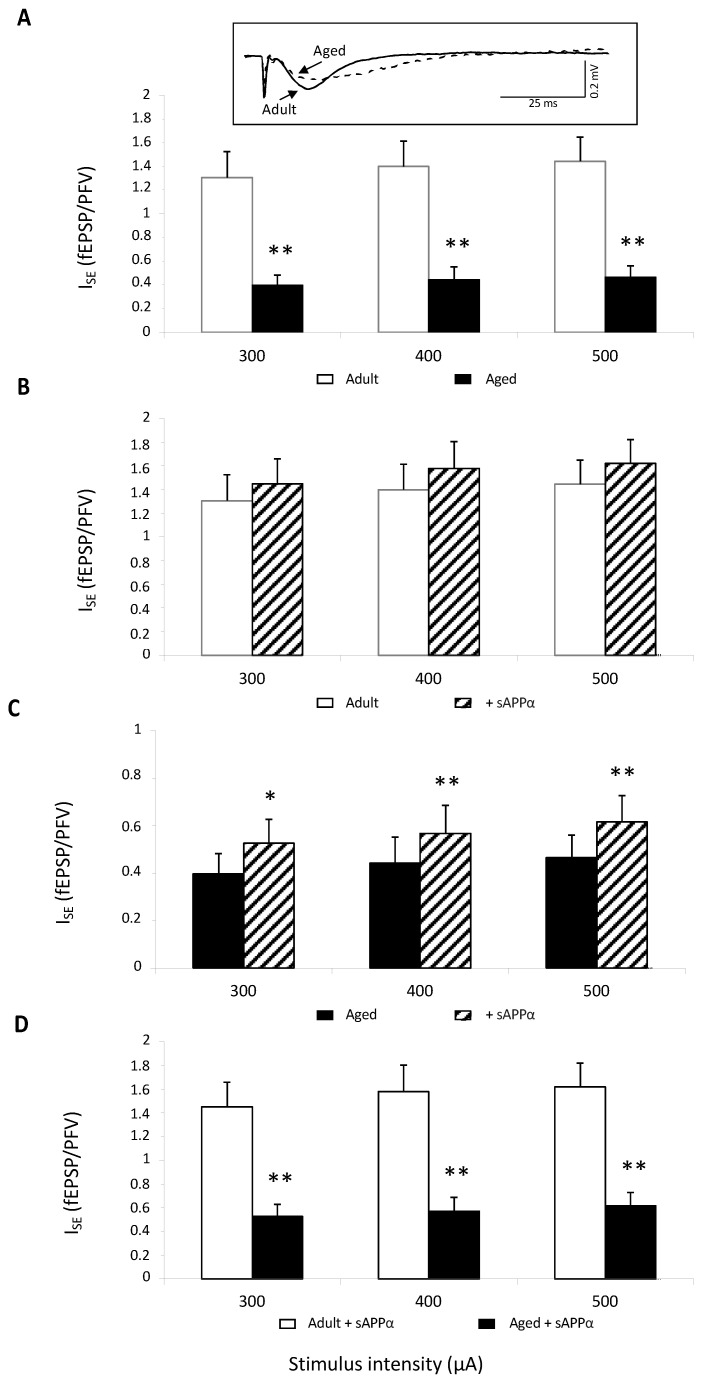
sAPPα (0.1 nM) improves NMDAR synaptic potentials in aged but not adult mice. (**A**) Comparison of the index of synaptic efficacy (I_SE_) corresponding to the fEPSP/PFV slope ratio of NMDAR-mediated synaptic potentials plotted against current intensity in 6 adult (n = 11 slices) and 6 aged mice (n = 11 slices) (** for *p* < 0.01 compared to adult mice one-way ANOVA). In the insert are superimposed representative traces of NMDAR-mediated fEPSPs obtained at 400 μA stimulation intensity. (**B**) Bar graphs illustrating the effects of sAPPα (0.1 nM) on I_SE_ of NMDAR-mediated fEPSPs in adult mice. (**C**) Bar graphs illustrating the effects of sAPPα (0.1 nM) on I_SE_ of NMDAR-mediated fEPSPs in aged mice (* and ** for *p* < 0.05 and *p* < 0.01, respectively, compared to control aCSF, paired *t*-test). (**D**) Comparison of the I_SE_ calculated in slices from adult and aged mice plotted against current intensity in sAPPα-supplemented aCSF (** for *p* < 0.01 compared to adult mice one-way ANOVA).

Isolated NMDAR-mediated synaptic potentials were first assessed. This was conducted in young adult and aged mice (11 slices from six animals in each group) by perfusing slices with a low-magnesium (Mg^2+^) artificial cortico-spinal fluid (aCSF) to relieve NMDARs from their Mg^2+^ blockade, and the aCSF was supplemented with the specific antagonist of non-NMDAR 2,3-dioxo-6-nitro-1,2,3,4-tetrahydrobenzoquinoxaline-7-sulfonamide (NBQX,10 μM) to isolate the NMDAR component. In these pharmacological conditions, stimulation of Schäffer collaterals induced a presynaptic fiber volley (PFV) followed by a long-lasting NMDAR-dependent field excitatory postsynaptic potential (fEPSP) (see the insert in Figure 1A). Regardless of the stimulus intensity, the index of synaptic efficacy (I_SE_) corresponding to fEPSP/PFV ratio was significantly reduced in aged compared to adult mice (age effect: F_1,20_ = 19.01, *p* = 0.0003), confirming the well-known age-related impairment of NMDAR activation (Figure 1A). Bath application of the recombinant sAPPα (0.1 nM) did not statistically affect I_SE_ in adult mice (Figure 1B), whereas a significant enhancement was induced in the aged group (15–20% increase in adult vs. 40% in aged mice regardless of stimulation intensity, Figure 1C). However, even though the sAPPα effect was significant only in aged mice, the age-related decrease in I_SE_ persisted (age effect: F_1,20_ = 19.69, *p* = 0.0003), indicating that the sAPPα delivery could only partially rescue the impairment of NMDAR activation (Figure 1D).

### 2.2. Theta-Burst-Induced Long-Term Potentiation (LTP)

Because NMDAR activation is critical for the expression of synaptic plasticity in neuronal networks [60,61,62,63], age-related effects of sAPPα on LTP expression were then investigated.

**Figure 2:**
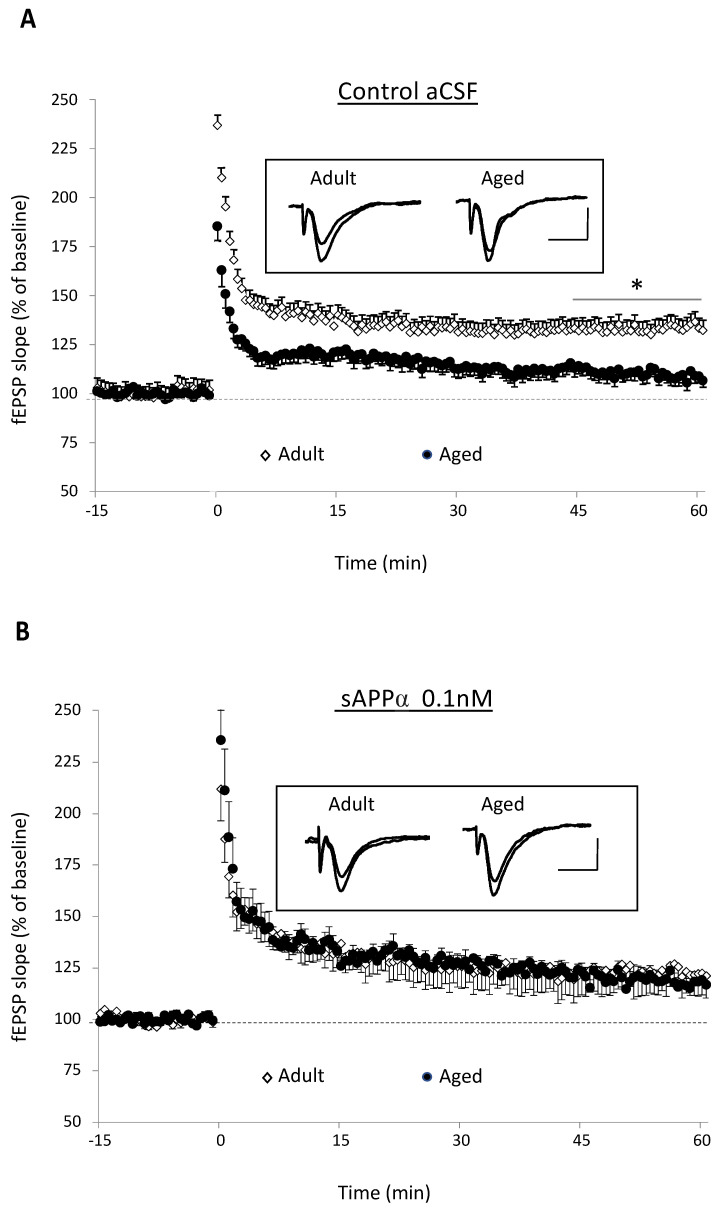
sAPPα rescues the age-related deficit of theta-burst-induced long-term potentiation. (**A**) Time-course comparison of theta-burst stimulation (TBS)-induced LTP in adult (10 slices/7 mice) and aged (12 slices/8 mice) animals in the control aCSF (* for *p* < 0.05 compared to adult mice two-way ANOVA in 15 last minutes and Bonferroni multiple comparisons test). In the insert are superimposed traces of fEPSPs recorded before and 60 min after TBS in an adult and aged mouse. (**B**) Time-course comparison of TBS-induced LTP in adult (10 slices/5 mice) and aged (10 slices/7 mice) mice in aCSF supplemented with sAPPα (0.1 nM). In the insert are superimposed traces of fEPSPs recorded before and 60 min after TBS in an adult and aged mouse (bars: 20 ms and 0.5 mV).

In 10 slices from seven adult mice, theta-burst stimulation (TBS) of glutamate afferents induced a significant and long-lasting increase in synaptic transmission (Figure 2A) averaging 33.1 ± 9.6% when calculated from the last 15 min of recording corresponding to the period of stable potentiation (stimulation effect: F_1,18_ = 13.32, *p* = 0.002). On the contrary, the same stimulation paradigm delivered in 12 slices from eight aged mice elicited only a weak (9.9 ± 3.9%) although statically relevant potentiation (stimulation effect: F_1,22_ = 8.44, *p* = 0.01) that, however, appeared significantly weaker than in adult mice (age effect: F_1,2_ = 6.66, *p* = 0.018, Figure 2A). These results confirm that TBS-induced LTP is impaired in the course of aging.

In recombinant sAPPα-supplemented aCSF, the mean potentiation induced by TBS in 10 slices from five adult mice (23.3 ± 10.6%) was not statistically altered compared to control medium, whereas it was significantly enhanced (19.1 ± 4.4%) when averaged in 10 slices from seven aged animals (drug effect: F_1,20_ = 24.9, *p* < 0.0001). Consequently, the mean LTP increase determined in the presence of the peptide was no longer different between adult and aged mice (age effect: F_1,20_ = 0.15, *p* = 0.69), indicating that sAPPα was able to rescue the age-related decrease in TBS-induced LTP (Figure 2B).

### 2.3. Basal Neurotransmission

**Figure 3:**
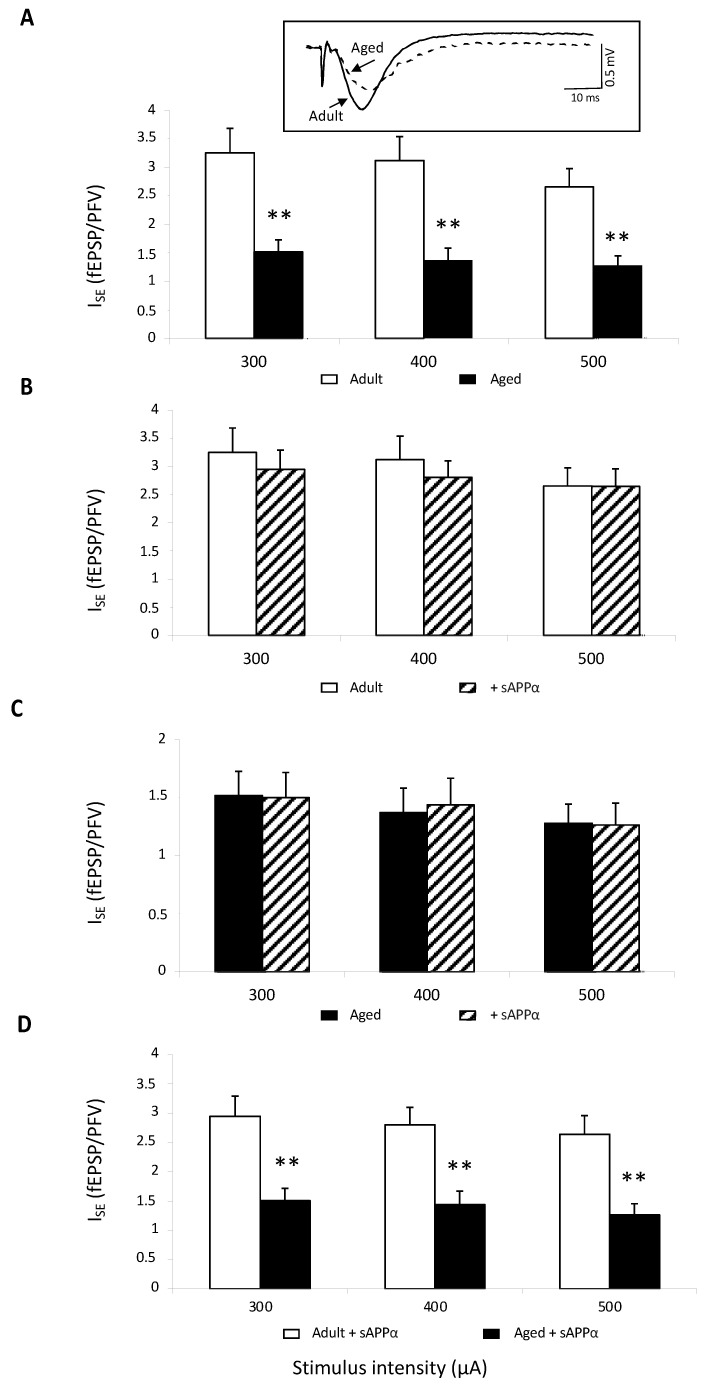
Basal neurotransmission is not affected by sAPPα. (**A**) Comparison of the index of synaptic efficacy (I_SE_) corresponding to the fEPSP/PFV slope ratio of non-NMDAR-mediated synaptic potentials plotted against current intensity in adult (12 slices/7 mice) and aged animals (11 slices/8 mice) (** for *p* < 0.01 compared to adult mice one-way ANOVA). In the insert are superimposed representative traces of non-NMDAR-mediated fEPSPs obtained at 400 μA stimulation intensity. (**B**) Bar graphs illustrating the effects of sAPPα (0.1 nM) on I_SE_ of nonNMDAR-mediated fEPSPs in adult mice. (**C**) Bar graphs illustrating the effects of sAPPα (0.1 nM) on I_SE_ of non-NMDAR-mediated fEPSPs in aged mice. (**D**) Comparison of the I_SE_ calculated in slices from adult and aged mice plotted against current intensity in sAPPα-supplemented aCSF (** for *p* < 0.001 compared to adult mice one-way ANOVA).

In order to determine whether the recombinant sAPPα-related improvement was specifically linked to NMDAR and not to a general glutamate deregulation, the effect of the peptide on basal glutamate neurotransmission was investigated in control aCSF. In these conditions in which NMDA-R activation is blocked by Mg^2+^ and only non-NMDA-R are activated, I_SE_ comparison showed a significant decrease in synaptic transmission in 11 slices from eight aged mice compared to values determined in 12 slices from seven adult animals (age effect: F_1,15_ = 3.64, *p* = 0.005), regardless of the stimulus intensity (Figure 3A). This result further supports the view of impaired basal neurotransmission in aging. When the recombinant sAPPα was added to aCSF, I_SE_ was not statistically impacted neither in adult (Figure 3B) nor in aged mice (Figure 3C), and the age-related impairment remained (Figure 3D).

In addition, sAPPα had no effect on paired-pulse facilitation (PPF), an electrophysiological paradigm assessing the presynaptic release of glutamate [64,65] in which two electrical stimuli were delivered with a 30 ms interval. In adult mice, the PPF ratio was 1.37 ± 0.05 in the control versus 1.30 ± 0.06 in sAPPα supplemented aCSF (n = 11) and 1.29 ± 0.07 versus 1.27 ± 0.08 in aged animals (n = 11) (Figure S1). These results indicate that the recombinant sAPPα did not affect presynaptic mechanisms involved in glutamate release.

### 2.4. Isolated NMDAR Activation in SR-KO Mice

In order to determine whether D-serine could be involved in sAPPα-related NMDAR improvement, the effects of bath application of the peptide were investigated in slices from SR-KO mice deprived of the NMDAR coagonist. Because 0.1 nM of sAPPα did not significantly impact NMDAR synaptic potentials in adult mice, we raised the concentration of the peptide to 1 nM, considering that a dose-dependent increase in NMDAR activation by sAPPα (until 10 nM) has been previously reported in rats [47,54,55].

**Figure 4:**
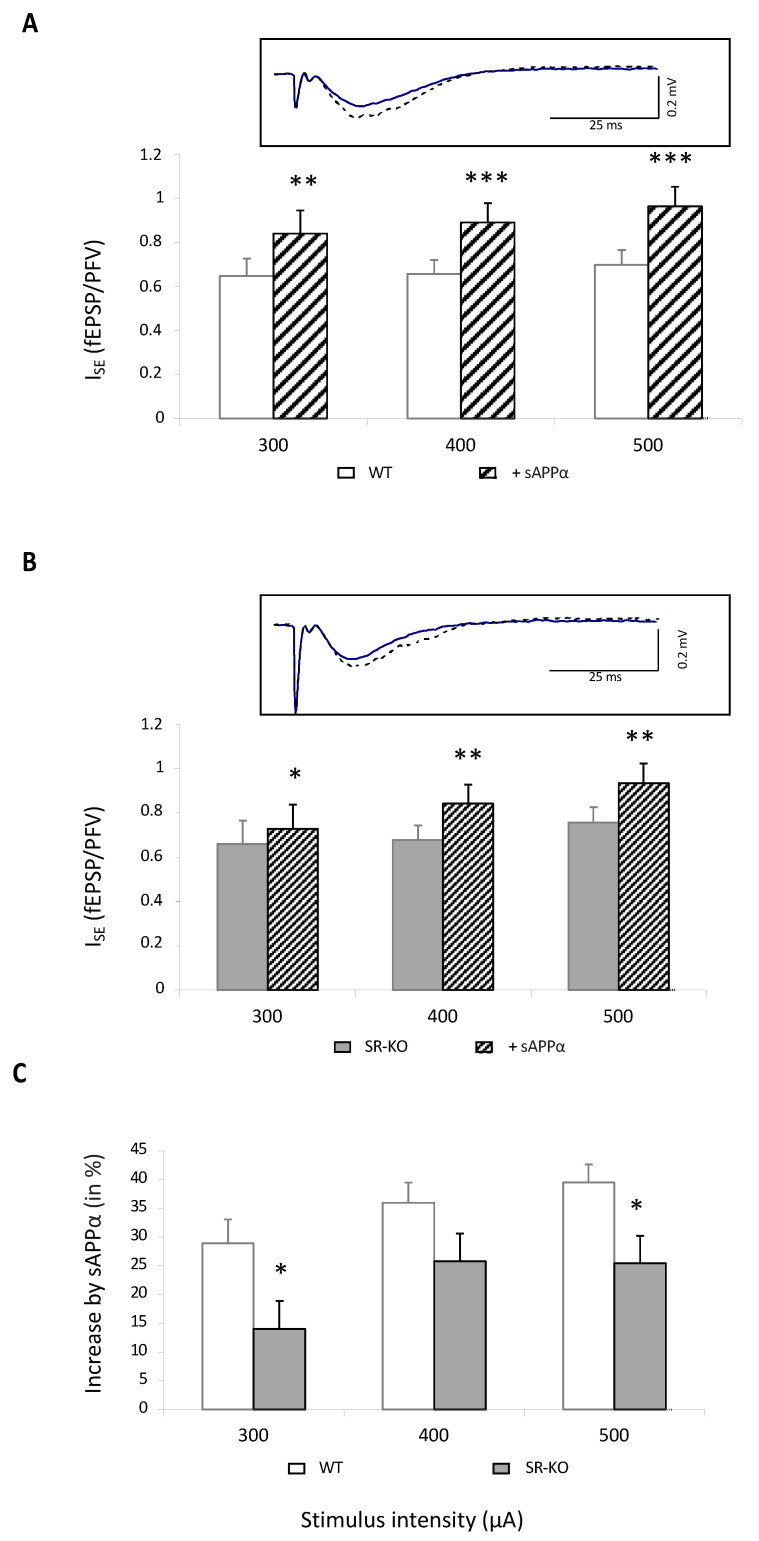
sAPPα-related improvement of NMDAR activation is lowered in SR-KO mice. (**A**) Bar graphs illustrating the effects of sAPPα (1 nM) on I_SE_ of NMDAR-mediated fEPSPs in 14 slices from 5 WT mice (** and *** for *p* < 0.01 and *p* < 0.0001, respectively, compared to control aCSF, paired *t*-test). In the insert are superimposed representative traces obtained at 400 μA stimulation intensity before (solid line) and after (dotted line) sAPPα supplementation. (**B**) Bar graphs illustrating the effects of sAPPα (1 nM) on I_SE_ of NMDAR-mediated fEPSPs in 20 slices from 8 SR-KO mice (* and ** for *p* < 0.05 and *p* < 0.01, respectively, compared to control aCSF, paired *t*-test). In the insert are superimposed representative traces obtained at 400 μA stimulation intensity before (solid line) and after (dotted line) sAPPα supplementation. (**C**) Comparison of the percent increase in NMDAR-related I_SE_ by sAPPα calculated in slices from WT and SR-KO mice plotted against current intensity (* for *p* < 0.05 compared to adult mice one-way ANOVA).

In 14 slices from five wild-type mice, NMDAR-related I_SE_ was significantly enhanced by 40%, regardless of stimulation intensity (Figure 4A). Interestingly, it was also statistically increased in 20 slices from eight SR-KO mice (Figure 4B) but only by 25% and therefore significantly less than in control animals (Figure 4C). These results therefore indicate that part of the improving effect of sAPPα on NMDAR activation involves the D-serine-related pathway.

## 3. Discussion

This study provides new complementary information on mechanisms underlying the beneficial property of sAPPα, the secreted form of the amyloid protein precursor generated by the non-amyloidogenic pathway, to prevent functional NMDAR synaptic deregulation underlying cognitive aging [42]. With the help of extracellular recordings at CA3/CA1 synapses of hippocampal slices, we first confirmed in mice that sAPPα is more efficient at improving NMDAR activation in aged than in adult animals and is thus able to rescue the LTP impairment thought to underlie age-related memory disability. In addition, we show that the sAPPα-induced NMDAR improvement is severely attenuated when serine racemase, which produces the NMDAR coagonist D-serine, is removed, suggesting this enzyme as one target for sAPPα.

Given the continuous aging of the world population, the question to prevent the degradation of cognitive abilities linked to age, and, notably, of memory capacities, is of ever-increasing importance that requires constant therapeutic research. Several decades of preclinical investigations have shown that cognitive impairment associated with physiological or pathological aging is the result of functional deregulation in brain neural networks [66,67,68]. The NMDAR subtype of glutamate receptors is critical for the expression of functional plasticity at synapses such as long-term potentiation, which is viewed as the molecular basis of memory formation [62,69,70,71,72], and data have accumulated to show that the expression and/or activity of NMDARs are impacted by age [8,11,12,73]. As such and because NMDAR modulation involves a mosaic of mechanisms, [74], they represent particular interest for researches seeking to offset age-related cognitive decline. In this context, it has been reported that sAPPα generated by the non-amyloidogenic pathway of the amyloid protein precursor promotes LTP facilitation [49,51,55,75,76,77]. In addition, this APP fragment could rescue memory deficits and impaired LTP in aged rats [46,47,54,55], but whether NMDAR contributes to the underlying molecular processes has yet to be defined [54,55,56]. In fact, sAPPα initiates alternative mechanisms such as activation of nicotinic α7-nACh receptors [50,51] or changes in glutamate receptor trafficking and protein synthesis [76,77] that could account for beneficial effects.

In the present study, we first show that sAPPα delivery facilitates NMDAR activation in adult mice, although with species differences, since threshold active concentration is higher than in rats (see [54,55]). Second, our recordings indicate that the beneficial effect of the APP fragment on NMDAR is larger in aged than in adult mice, and that it consequently rescues the age-related impairment of functional plasticity at CA3/CA1 hippocampal synapses (see also [47,54] for aged rats). Finally, we show that the improving effect of sAPPα is blunted in SR-KO mice with a 90% decrease in D-serine levels [15,78,79], thus providing functional evidence for intrinsic NMDAR contribution in mechanisms of the facilitation. Indeed, NMDAR activation requires the binding of the coagonist D-serine on glycine sites present on GluN1 subunits concomitantly to the presence of glutamate on GluN2 subunits [3,74]. The weak effect of sAPPα in SR-KO mice therefore strongly suggests that the APP fragment promotes an increase in D-serine availability at synapses that, in addition to enhancing surface expression of GluN2B subunits [77], facilitates NMDAR activation and LTP expression. Such mechanisms could also explain why sAPPα is more efficient in aged animals. Indeed, hippocampal aging is associated with a weaker occupancy of NMDAR glycine binding sites due to a decrease in D-serine levels [21,80,81] and a reduction of GluN2B subunits expression [82,83,84,85,86].

Different mechanisms may account for an increase in D-serine by sAPPα. It is reported that short- but not long-term exposure of rat hippocampal organotypic slice cultures to sAPPα elicits upregulation of many immediate early gene transcription factors, including AP-1 [87], which targets the promoter of the D-serine synthesizing enzyme SR [88]. Accordingly, transcriptional SR expression is promoted by the APP fragment that is associated with D-serine release [57], though these effects have, as of yet, only been characterized in microglia cultures. Alternatively, increased oxidation [89] and/or changes in dimer active conformation [90] by changes in redox status are thought to alter SR activity, especially in the aging brain in which potent oxidative stress occurs (see [4]). Considering that sAPPα activates signaling pathways that protect synapses against excitotoxicity linked to increased oxidation and impaired energy metabolism [91,92,93,94], this antioxidative property may help sAPPα to optimize SR activity and D-serine production.

Our study provides additional information to consider the manipulation of the NMDAR gating process by endogenous D-serine as a pertinent and selective strategy to prevent synaptic deregulation driving age-related cognitive defects. A similar conclusion was recently reached with D-isoleucine (D-Ile), which stimulates the activity of the Asc-1 subtype of neutral amino acid transporters, promoting neuronal D-serine release [95]. Indeed, D-Ile delivered to hippocampal slices from aged rats also rescued LTP deficits [20]. Because D-serine is specified as a major endogenous NMDAR coagonist mainly in cognitive-associated brain structures [15,16,17,18,19], its manipulation is therefore expected to help in improving solely related functions such as learning and memory without interfering with other NMDAR-dependent processes. In addition, it is worth notice that altering endogenous D-serine does not affect basal synaptic transmission mediated by the other subtypes of glutamate receptors, which also lowers the impact of the pharmacological manipulation on disturbing the overall brain activity. This is in contrast with the use of the partial agonist D-cycloserine (DCS) acting at NMDAR glycine-binding sites, which also rescues age-related defective memory function and impaired LTP [96,97,98]. Indeed, DCS at the same time reduces basal synaptic communication and enhances intrinsic neuronal excitability [99,100]. These adverse synaptic effects could explain why DCS delivery also promotes unexpected behavioral responses such as generation of episodic-like memory [101] or altered long-term memory consolidation [102].

## 4. Materials and Methods

All experiments were carried out in accordance with the European Communities Council Directive (63/2010) regarding the care and use of animals for experimental procedures and approved by the local ethical committee. The experiments were conducted with young adult (4–6 months) and aged C56Bl7 male mice (24–25 months) purchased from Janvier Labs (France), while young adult (4–6 months) SR-KO mice also with C56Bl7 background were kindly provided by Pr. H. Wolosker (Technion Institute of Technology, Israel). Mice were housed five to a Plexiglas cage and maintained on a controlled light–dark cycle, with constant temperature (22 ± 2 °C) and ad libitum access to food and water.

### 4.1. Hippocampal Slices Electrophysiology

Transverse hippocampal slices (400 µm) were prepared in aCSF and placed in a holding chamber for at least 60 min. The composition of aCSF was (in mM): NaCl 124, KCl 3.5, MgSO_4_ 1.5, CaCl_2_ 2.3, NaHCO_3_ 26.2, NaH_2_PO_4_ 1.2 and glucose 11 (pH 7.4). A single slice was transferred to the recording chamber and continuously submerged with aCSF pre-gassed with 95%O_2_/5%CO_2_ mixture.

Extracellular recordings were obtained at room temperature from the apical dendritic layer of the CA1 area using glass micropipettes (2–5 MΩ) filled with 2 M NaCl. Presynaptic fiber volleys (PFVs) and non-N-methyl-D-aspartate receptor (NMDAR)-mediated field excitatory postsynaptic potentials (fEPSPs) were evoked at 0.1 Hz by electrical stimulation of Schaffer collaterals and commissural fibers located in the stratum radiatum to assess basal neurotransmission. The averaged slope of three PFVs and fEPSPs was measured using Win LTP software (WinLTP Ltd., Bristol, UK) [103,104]. To evaluate the level of synaptic efficacy at CA3/CA1 synapses, an index (I_SE_) corresponding to fEPSP/PFV ratio was calculated. PFV and fEPSP slopes as well as I_SE_ were plotted against stimulus intensity (300, 400 and 500 µA).

Paired-pulse facilitation (PPF) of basal synaptic transmission was induced by electrical stimulation of afferent fibers with paired pulse (interstimulus interval of 30 ms). PPF was calculated as the ratio of the slope of the second response over that of the first one.

Specific NMDAR-mediated fEPSPs were isolated in slices perfused with low-Mg^2+^ (0.1 mM) aCSF containing the non-NMDAR (AMPA) antagonist 2,3-dioxo-6-nitro-1,2,3,4-tetrahydrobenzoquinoxaline-7-sulfonamide (NBQX, 10 µM). Again, the fEPSP/PFV ratio was plotted against stimulus intensity (300, 400 and 500 µA) to assess the level of NMDAR activation.

Long-term potentiation (LTP) of synaptic transmission was investigated using theta-burst stimulation (TBS) paradigm. A test stimulus (0.1 Hz) was adjusted to obtain an fEPSP with a baseline slope of 0.1 V/s. The slope of three averaged fEPSPs was measured for 15 min before TBS delivery. The conditional stimulation consisted of 5 trains of four pulses at 100 Hz separated by 200 ms. This sequence was repeated three times with an interburst interval of 10 s. Testing with a single pulse was then resumed for 60 min after the conditioning stimulation to determine the level of stable potentiation.

### 4.2. Preparation of Recombinant sAPPα

Recombinant sAPPα was kindly provided by Drs. C. Rose and B. Allinquant and prepared as previously described [105]. A DNA fragment harboring the coding sequence for sAPPα was first generated by PCR amplification of a plasmid encoding for human APP695. The forward primer (Eurofins MWG Operon, Ebersberg, Germany) was 5′-ACTGTCGACTATGCTGCCCGGTTTGGCA-3′ containing a Sal1 restriction site, and the reverse primer was 5′-CAGCGGCCGCTTTTTGATGATGAACTTC-3′ containing a Not1 restriction site. The amplified DNA were cloned into pGEX-6P-2 (GE Healthcare, Buc, France) containing a PreScission Protease sequence upstream to the Sal1 restriction site for cleaving the GST tag from the fused protein. The generated plasmids were sequenced.

The plasmid was then transformed into an *E. coli* strain BL21pLysS (Invitrogen, ThermoFisher scientific, Waltham, MA, USA). The transformed cells were grown in 25 mL of YT broth medium containing 100 µg/mL ampicilline at 37 °C for 16 h then diluted 1/10 in the same medium and grown to an absorbance of 0.3 at 600 nm. Proteins encoded by the plasmids were induced by addition of isopropyl-β-D-thiogalactopyranoside (IPTG) to achieve a final concentration of 0.1 mM, which was then incubated for an additional 3 h at 20 °C. The bacteria were then collected by centrifugation, resuspended in 5 mL of phosphate buffer saline (PBS) containing 0.2 mg/mL lysozyme and 5 mM dithiothreitol, and lysed with a sonicator 1 min at 4 °C. Triton X100 1% was added, and the mixture was stirred gently for 2 h at 4 °C and then centrifuged 15 min at 14,000× *g* at 4 °C. The supernatants were added to a glutathione–sepharose column equilibrated with binding buffer. After washings, proteins were eluted from the glutathione–sepharose beads with PreScission Protease (GE Healthcare) at 4 °C during 24 h, according to the manufacturer’s instructions. The recombinant protein was analyzed by SDS-PAGE for its respective MW and checked by immunoblot using the N-terminal APP antibody (MAB 348, Chemicon International, Sigma Aldrich Chimie, Sarl, Saint-Quentin Fallavier, France) and the C-terminal specific sAPPα antibody (6E10, Signet, Gentaur, France) that did not recognize the sAPPβ.

### 4.3. Pharmacology

In all electrophysiological experiments, the recombinant sAPPα was perfused at the final concentration of 0.1 nM or 1 nM for least 15 min before the start of the recording to ensure large diffusion of the peptide in tissues and full expression of the effects. NBQX required to isolate specific NMDAR-mediated synaptic potentials (ref 1044) was purchased from Tocris biosciences® (Noyal Chatillon sur Seiche, France).

### 4.4. Data Analysis

All results are expressed as the mean ± S.E.M. After control of data normality (Shapiro–Wilk test), one-way ANOVA was used to assess the significance of age-related changes in basal neurotransmission, PPF or isolated NMDAR activation, while sAPPα age-related effects were determined using paired *t*-tests. The significance of LTP expression was assessed by comparing the 15 min of baseline recordings with values recorded between 45 and 60 min after the TBS, while the sAPPα-related effects were estimated by comparing the last 15 min of recordings. *p* values were calculated using multivariate analysis of variance followed by Tukey’s post hoc tests (StatView software) to account for the correlations inherent to repeated measures. In all cases, differences were considered significant when *p* < 0.05.

## Data Availability

The data presented in this study are available on request from the corresponding author. The data are not publicly available because they belong to our funding.

## Supplementary Materials

The supporting information can be downloaded at: https://www.mdpi.com/article/10.3390/ijms232415542/s1.
