# Supplementary figures and images for: Improved NMDA Receptor Activation by the Secreted Amyloid-Protein Precursor-α in Healthy Aging: A Role for D-Serine?
Source: Int J Mol Sci. 2022 Dec 8;23(24):15542. doi: 10.3390/ijms232415542 (PMC9779005; doi:10.3390/ijms232415542)

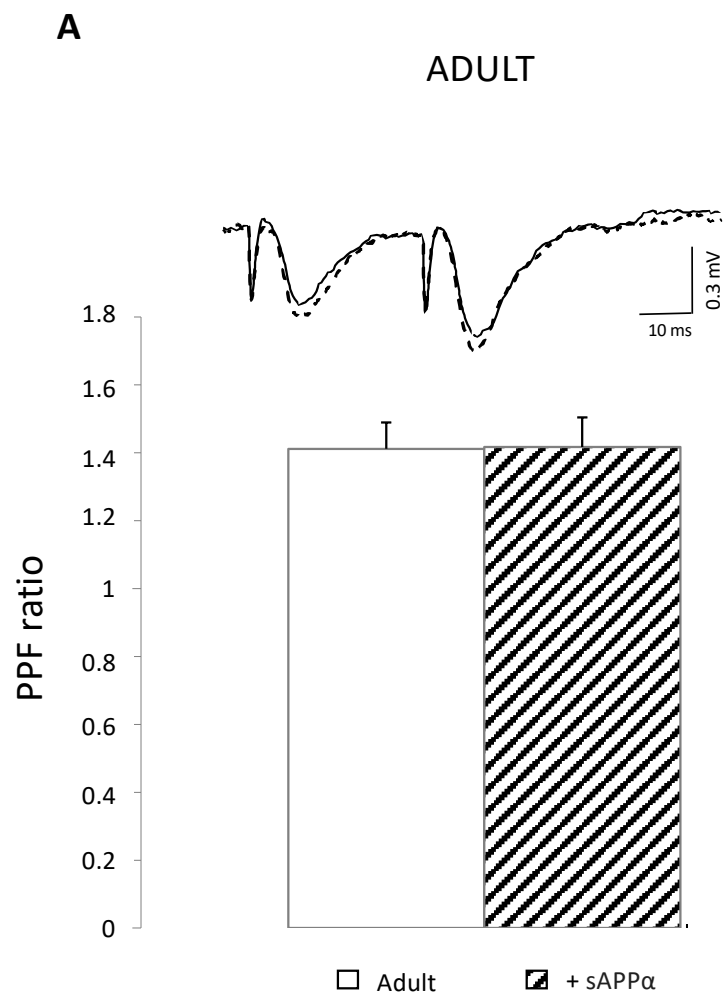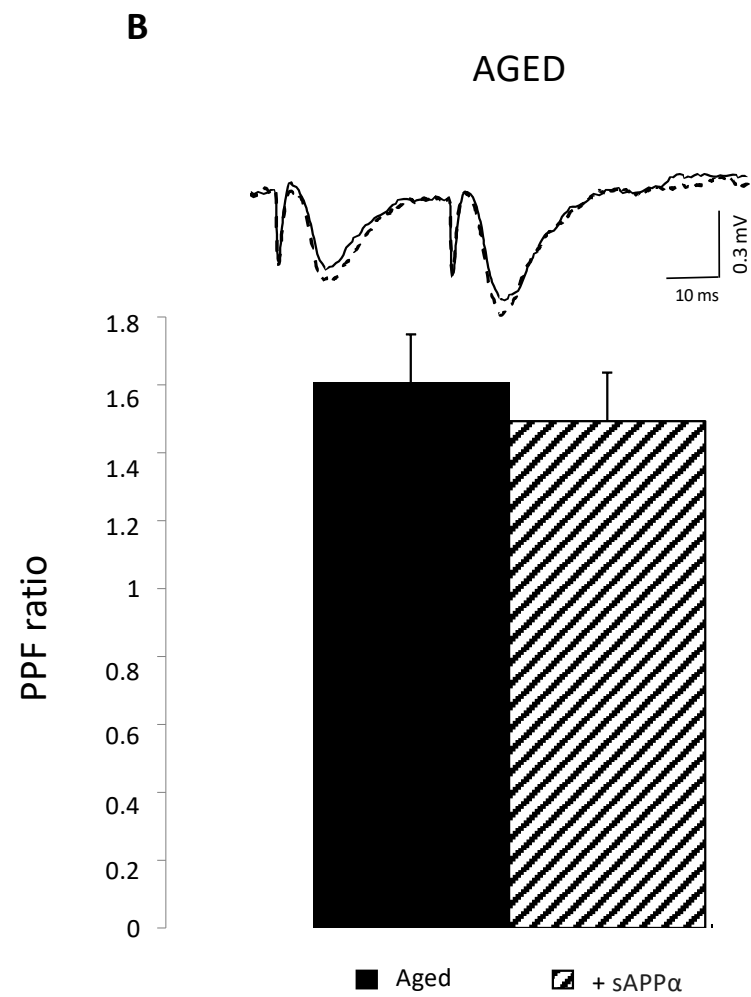

Supplement: Supplementary file 1 [file ijms-23-15542-s001.zip › ijms-2019525-supplementary.pdf]
